# Creating Solid Solutions of Metallocenes: Migration of Nickelocene into the Ferrocene Crystal Lattice in the Absence of a Solvent

**DOI:** 10.1021/acs.jpcc.2c07441

**Published:** 2023-02-06

**Authors:** Gabrielle E. Harmon-Welch, John C. Hoefler, Martha R. Trujillo, Nattamai Bhuvanesh, Vladimir I. Bakhmutov, Janet Blümel

**Affiliations:** Department of Chemistry, Texas A&M University, College Station, Texas 77845-3012, United States

## Abstract

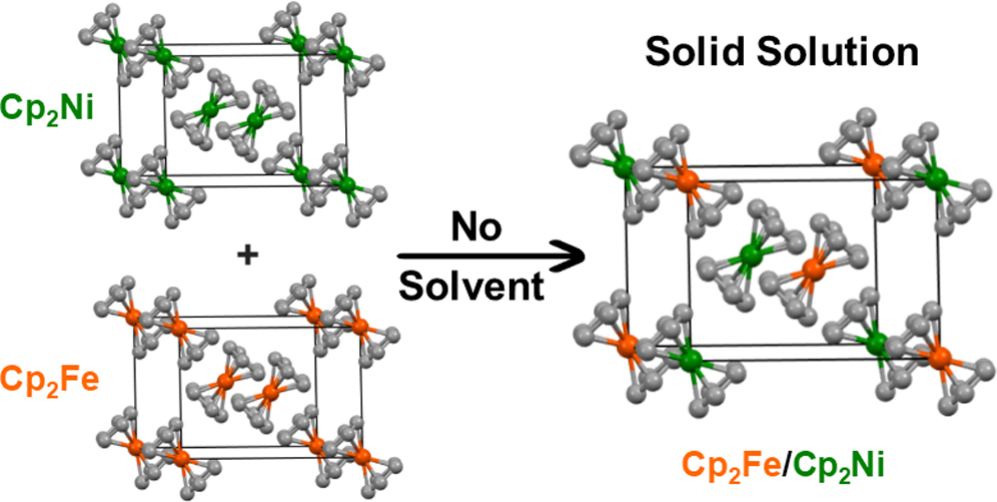

Ferrocene and nickelocene do not react with each other in solution; however, the large impact of the paramagnetic component on the ferrocene ^1^H NMR signal linewidth and relaxation times has been quantified. Co-crystallization of ferrocene and nickelocene at any ratio from a solvent can be explained with both pure substances crystallizing in the same space group *P*2_1_/*n*. As a new phenomenon, when a ferrocene single crystal is exposed to polycrystalline nickelocene in the absence of a solvent, the nickelocene migrates into the ferrocene crystal lattice and a mixed crystal is formed that retains its macroscopic shape. This process has been proven visually by cutting the single crystal. Mixing polycrystalline ferrocene with polycrystalline nickelocene at different molar ratios with a mortar and pestle leads to crystalline solid solutions with the corresponding molar ratios of both components. This migration of one organometallic component into an existing crystal lattice of another at ambient temperature in the absence of a solvent has not been described previously. Paramagnetic ^1^H solid-state NMR spectroscopy of static and rotating samples of dry ferrocene/nickelocene mixtures at varying ratios is used to prove and quantify the mixing of both metallocenes at the molecular level. A single-crystal X-ray structure of a 50/50 mixed crystal corroborates the NMR results that nickelocene and ferrocene are randomly distributed in the lattice and that the space group *P*2_1_/*n* is retained. All ferrocene molecules in the mixed crystal lattice show a broadening of their ^1^H wideline signals and residual magic-angle spinning (MAS) lines at ambient temperature. The broadening of the ferrocene signals correlates with the nickelocene content. ^1^H *T*_1_ relaxation time measurements for the signals of ferrocene in samples with different amounts of nickelocene corroborate the assumption that the signal broadening is due to paramagnetic dipole–dipole relaxation of ferrocene molecules in the vicinity of nickelocene. Spatially separated ferrocene and nickelocene powders in one rotor show the solid-state NMR characteristics of the individual polycrystalline metallocenes. The described formation of solid solutions of metallocenes in the absence of a solvent will open new pathways to homogeneously mixed nanoparticles with desired metal ratios and dual-atom catalysts.

## Introduction

Co-crystallization for the purpose of generating solid solutions^1^ is an important technique to create mixed materials with well-defined contents and spatial distribution of different components.^2^ In particular, pharmaceutical chemistry depends on solid solutions, e.g., medicine homogeneously distributed in a polymer matrix, and co-crystallized materials.^3^ These can be generated by crystallizing the components from optimized solvent mixtures. Alternatively, in case the substances are sufficiently volatile, the co-crystallized matter can be created from condensing mixed vapors under well-defined conditions.^4^

Interestingly, co-crystallization or the formation of solid solutions of crystalline metallocenes has not been described in the literature yet, although such well-defined materials would have great potential as precursors for creating heterobimetallic nanoparticles and dual-atom catalysts.^5^ In particular, ferrocene and nickelocene are interesting targets for co-crystallization because they prefer the same space group *P*2_1_/*n* for their single crystals and have practically the same size and shape.^6,7^ Earlier, we have studied ferrocene and nickelocene, adsorbed together on a silica surface, and found that they mix on the molecular level.^8^ This mixing is easily explained by the dynamics and especially the translational mobility of both metallocenes on the surface.^9−14^ As a next step, we sought to investigate mixtures of pure ferrocene and nickelocene regarding their potential to form randomly mixed crystals with homogeneous distribution of the components in the crystal lattice, i.e., crystalline solid solutions of the metallocenes.

In this study, we confirm that indeed, ferrocene (Cp_2_Fe, (C_5_H_5_)_2_Fe, **1**)^6^ and nickelocene (Cp_2_Ni, (C_5_H_5_)_2_Ni, **2**)^7^ form crystalline solid solutions at any ratio when both components are allowed to crystallize together from a solvent. Alternatively, the solvent can be removed from the solutions of the combined metallocenes to obtain solid solutions. Most importantly, a new phenomenon, the migration of nickelocene into a ferrocene crystal lattice in the absence of a solvent, will be described. The distribution of the components in the crystal lattice is demonstrated visually and investigated by ^1^H solid-state NMR spectroscopy^8−16^ and *T*_1_ relaxation time measurements.^17^

## Experimental Section

### Sample Preparation

Nickelocene and ferrocene were obtained commercially and purified by sublimation under inert gas prior to use. Large single crystals of ferrocene were grown from saturated hexane solutions. For creating the nickelocene/ferrocene solid solutions within minutes without solvents, the polycrystalline components were ground together with a mortar and pestle in a glovebox in the corresponding molar ratio. All samples were densely packed into the rotors as finely ground powders. Compressed nitrogen was used as both the bearing and drive gas for the magic angle spinning (MAS) measurements.

### NMR Measurements

The solution NMR measurements were recorded on a Varian 500 MHz instrument at ambient temperature in CDCl_3_. The ^2^H and ^1^H MAS and static NMR experiments were performed using a Bruker Avance 400 solid-state NMR spectrometer (400 MHz for ^1^H nuclei) equipped with a two-channel 4 mm MAS NMR probe head. The standard single-pulse and Hahn-echo sequences were applied for ^1^H and ^2^H nuclei, respectively. The Hahn-echo experiments were synchronized with spinning rates. Typically, eight scans were sufficient for obtaining spectra of high quality, and no line broadening had to be applied for processing. The ^1^H background signal was recorded under identical conditions and subtracted from the spectra of the samples.

The ^1^H *T*_1_ times were measured at a rotational frequency of 10 kHz using inversion-recovery (180°−τ–90°) experiments with well-calibrated radio frequency (RF) pulses, τ variations between 0.0005 and 50 s, and with relaxation (recycle) delays corresponding to complete relaxation of the nuclei after each pulse cycle. The experimental inversion-recovery curves (signal intensity versus τ time) have been treated with a standard nonlinear fitting computer program based on the Levenberg–Marquardt algorithm. In the mixed systems due to the large chemical shift difference for **1** and **2**, the relaxation times were obtained at two carrier frequencies centered at the positions of both resonances.

The processing to determine the ^1^H sideband intensity values and the line-shape analyses of the static ^1^H NMR spectra were performed with the corresponding programs in the software package of the Bruker spectrometer.

## Results and Discussion

In solution, ferrocene and nickelocene do not undergo chemical reactions. At different molar ratios, chloroform solutions gradually change color from bright orange for pure **1** to blue-green for **2** (Figure S1), without forming a precipitate. The solutions with varying contents of **1** and **2** can easily be studied by paramagnetic NMR spectroscopy.^9,10,17,19−25^ The ^1^H NMR spectrum of one example is shown in Figure S2. The paramagnetic nickelocene signal at about −252 ppm is much broader than that of the diamagnetic ferrocene.^24^ However, the influence of **2** on **1** in solution is noticeable. The ^1^H NMR chemical shift and linewidth data are summarized in Table S1. The chemical shift of the ferrocene resonance increases from 4.16 for pure **1** to 4.90 for a 10/90 molar ratio with **2**. The half-width of the signal of **1** increases accordingly from 2.0 to 5.5 Hz. Since nickelocene is not a chemical shift reagent, coordinating or interacting otherwise with **1**, the effect on the chemical shift and linewidth of the signal of **1** is limited. The ^1^H NMR signal of nickelocene remains practically unchanged at all molar ratios with half-widths between 540 and 560 Hz (Table S1).

Next, the ^1^H *T*_1_ relaxation times of the signals of **1** and **2** in different mixtures were obtained using inversion-recovery techniques.^17^ The data are summarized in Table S2. The *T*_1_ time of the nickelocene signals in the various mixtures has a value of roughly 1 ms, which remains remarkably consistent at all ratios. However, the ferrocene protons relax much faster when the content of nickelocene is high. From initially 4.9 s for pure ferrocene in chloroform, with increasing amounts of nickelocene, the *T*_1_ time decreases to about 180 ms for a 10/90 molar ratio of **1** and **2**. Therefore, it can be concluded that in solution, the effect of the paramagnetic nickelocene on the ferrocene signal is more pronounced in the longitudinal relaxation of **1** than its chemical shift or linewidth.

**Figure 1:**
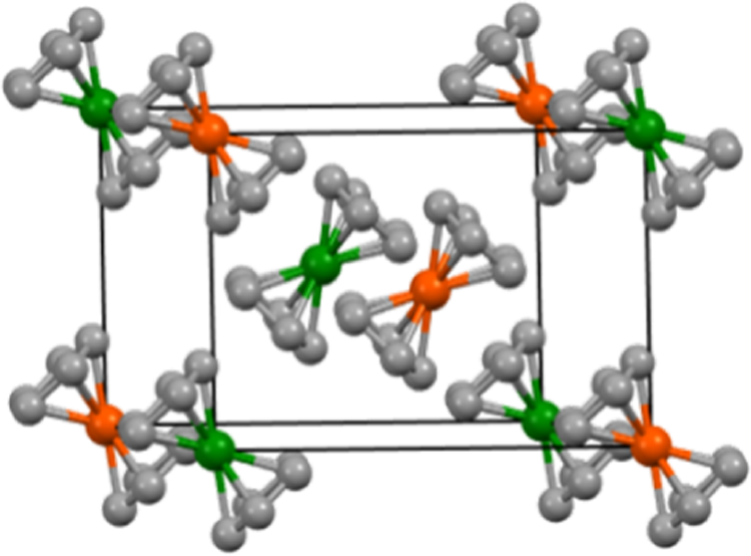
Schematic display of a unit cell of a mixed ferrocene/nickelocene crystal (molar ratio 50/50) obtained from solution. The unit cell was created using the data of the single-crystal X-ray structure of ferrocene.^18^

When ferrocene and nickelocene are dissolved together in hexane and allowed to crystallize, solid solutions form. The color of the crystals differs from the orange of the pure ferrocene and the dark green of nickelocene. Depending on the initial amounts of the components, the crystals range in color from orange for a 90% ferrocene/10% nickelocene mixture to dark green for 10% **1** and 90% **2**. The schematic unit cell of a solid solution of ferrocene/nickelocene is displayed in Figure 1. Mixtures of the polycrystalline components range from yellow to lime green.

The ratios of **1** and **2** in the mixed crystals can be determined by decanting the supernatant liquid, redissolving the isolated crystals, and recording their ^1^H NMR spectra in solution. The ratios of **1** and **2** in the mixed crystals correspond to the ratios of the components dissolved prior to crystallization. This means that there is no preference for molecules of **1** or **2** in the mixed crystal lattice. It is noteworthy to point out that the ratios of **1** and **2** in the obtained crystals could in principle also be determined by ^1^H MAS NMR of the crystals. However, as described below, the precision of the integration suffers from the presence of the proton background signal in the solid-state measurements that overlaps with the ferrocene resonance (see below). Nevertheless, after background subtraction, the integration gave ratios similar to those in Table 1 within the error limits of 12–15%.

**Table 1:** ^1^H MAS NMR Half-Widths Δν_**1**/**2**_ of the Isotropic Lines of Ferrocene (**1**) and Nickelocene (**2**) Signals in a Solid Mixture of the Polycrystalline Components with the Indicated Molar Ratios at a Spinning Speed of 10 kHz

molar ratio **1**/**2** (%)	Δν_**1**/**2**_ of **1** (kHz)	Δν_**1**/**2**_ of **2** (kHz)
100/0	0.79	
90/10	0.86	2.77
80/20	0.92	2.49
70/30	0.99	2.50
60/40	1.07	2.33
50/50	1.18	2.58
40/60	1.31	2.36
30/70	1.52	2.46
20/80	1.53	2.49
10/90	1.52	2.47
0/100		2.57

Iron and nickel atoms have similar numbers of electrons (26 and 28), making the task to distinguish both atoms by X-ray diffraction difficult. Nevertheless, a mixed ferrocene/nickelocene crystal has been investigated.^26^ For this purpose, a single crystal of high quality has been grown from a 50/50 solution of ferrocene and nickelocene in pentane. Most importantly, the space group *P*2_1_/*n* of the mixed crystal remains the same as that of pure ferrocene and pure nickelocene.^6,7^ Since the data quality of this X-ray structure is very good, two distinct orientations of the Cp rings are clearly visible. Assuming that the crystal was composed of pure nickelocene, the occupancy of nickel atoms decreased when the data were refined. This indicates disorder with an element other than nickel. On the other hand, assuming that iron atoms take the place of nickel, the iron occupancy is increasing upon refinement. Overall, the refinement operations resulted in occupancies of ferrocene and nickelocene close to a 50:50 ratio. This corroborates the results of the solid-state NMR measurements outlined below. There is no indication of a unit cell doubling in the X-ray structure, and there are no well-defined super-structures in the crystal. This means that iron and nickel atoms are not ordered within the crystal but rather disordered and randomly distributed.

Measuring the ^1^H NMR spectra of the mixed crystals with a large sweep width proved that **1** and **2** do not react with each other. In particular, no ferrocenium ion signals with chemical shifts of about 330 ppm, and no impurities in the diamagnetic region that would indicate any metallocene decomposition, were visible. As a check, when the mixtures and solutions of pure nickelocene are exposed to air, the ^1^H NMR spectra indicate the formation of cyclopentadiene and the Diels–Alder addition product dicyclopentadiene.^27^

Setting the stage for our next investigation, we determined the melting points of **1** and **2** (Table S3). The literature-known melting range for **1** could be verified (172–174 °C; reference: 172.5 °C).^28^ Interestingly, however, the range for **2** (152–156 °C) differed substantially from the reported value (172 °C).^28^ Regardless, both melting points are relatively high, and grinding the polycrystalline components **1** and **2** together using a mortar and pestle at ambient temperature does not result in any melting process. Nevertheless, when **1** and **2** are ground together in the absence of a solvent, mixed crystals form within minutes. The change in the ratio of the components in the dry mixtures is clearly reflected in the changes in the melting points (Table S3). The distribution of the metallocenes within the crystal lattice is described below.

**Figure 2:**
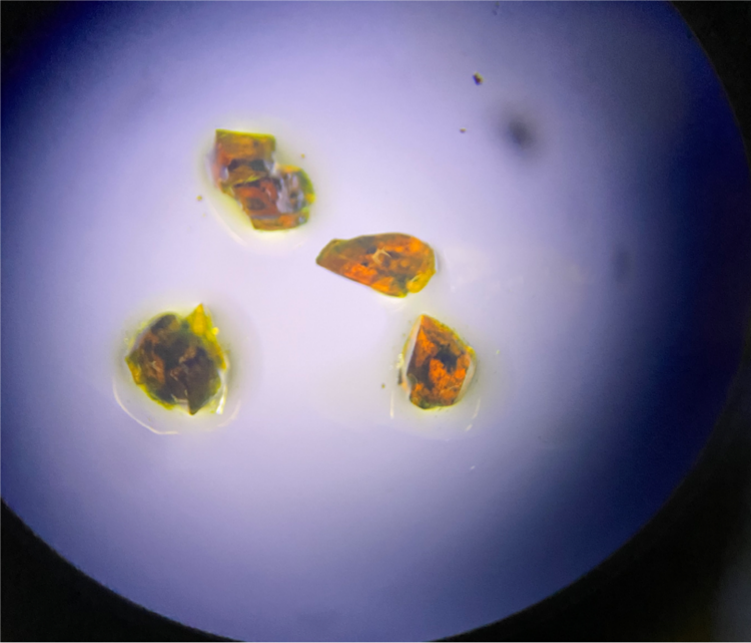
Specimens of mixed ferrocene/nickelocene crystals generated by allowing migration of nickelocene into ferrocene single crystals.

To confirm visually that nickelocene can indeed migrate into a ferrocene crystal lattice, single crystals of **1** have been grown and placed onto polycrystalline **2** (Figure S3). The originally orange crystals of **1** turned green within 24 h. After being exposed to the polycrystalline **2** for 10 weeks, representative ferrocene single crystals with ca. 4 mm diameter were cut in half to explore whether the distribution of **2** in the crystal of **1** was homogeneous. As Figure 2 shows, nickelocene has migrated into the ferrocene crystal along pathways and then formed domains inside. The result is a marbled pattern.

**Figure 3:**
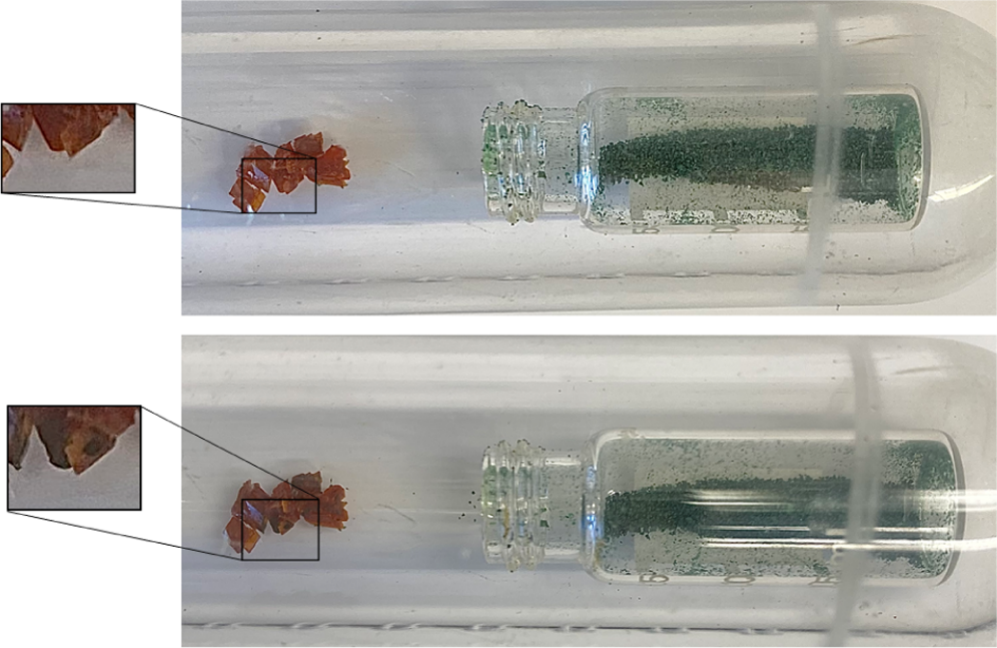
Specimens of mixed ferrocene/nickelocene crystals generated by allowing diffusion of nickelocene to ferrocene single crystals.

Next, we checked whether nickelocene can also migrate into single crystals of ferrocene from the gas phase and not only through direct contact. For this experiment, single crystals of **1** have been placed outside a vial with polycrystalline **2** under an inert gas atmosphere (Figures 3 and S4). In this case, the process is much slower than with direct contact of the crystals or mechanical mixing of powders. However, in the course of days, the volatile nickelocene darkened the ferrocene crystals in certain areas. Most probably, this is because **2** enters the single crystals of **1** at the points of crystal defects. Importantly, the color change is not limited to the surface but is again present throughout the crystals, indicating that nickelocene penetrates all of the ferrocene specimens.

To investigate whether the distribution of molecules of **1** and **2** is homogeneous throughout the crystal lattice and a genuine solid solution forms, solid-state NMR spectroscopy has been applied.^8−17,19−22,29−33^ As mentioned above, while **1** is diamagnetic, **2** is paramagnetic, featuring two unpaired electrons. The latter have an impact on the solid-state NMR spectra; however, paramagnetic NMR spectroscopy of solids is well established.^8−10,12,17,19−22,34,35^ In the case of a solid solution with random distribution of **1** and **2** in the crystal, the paramagnetism of **2** should influence the ^1^H NMR signal characteristics of **1**. For diamagnetic compounds and materials, besides the residual linewidth (half-width of the isotropic line when the sample is rotated), the chemical shift anisotropy (CSA, overall shape of the signal including the rotational sidebands)^29−31,35^ is a relevant characteristic of a functional group or material. While the residual linewidth of the ^1^H MAS signals of **1** can still be used as an indicator for the presence of molecules of **2** close to **1**, unfortunately, the CSA will be inextricably mingled with the broadening effects of dipolar interactions and the bulk magnetic susceptibility (BMS).^17^ Nevertheless, the changing signal shape of **1** with increasing amounts of **2** can be evaluated by the growing sideband intensities, as described below. Finally, the *T*_1_ relaxation time of the proton signal of **1** should decrease with increasing amounts of **2** in the crystal lattice.

**Figure 4:**
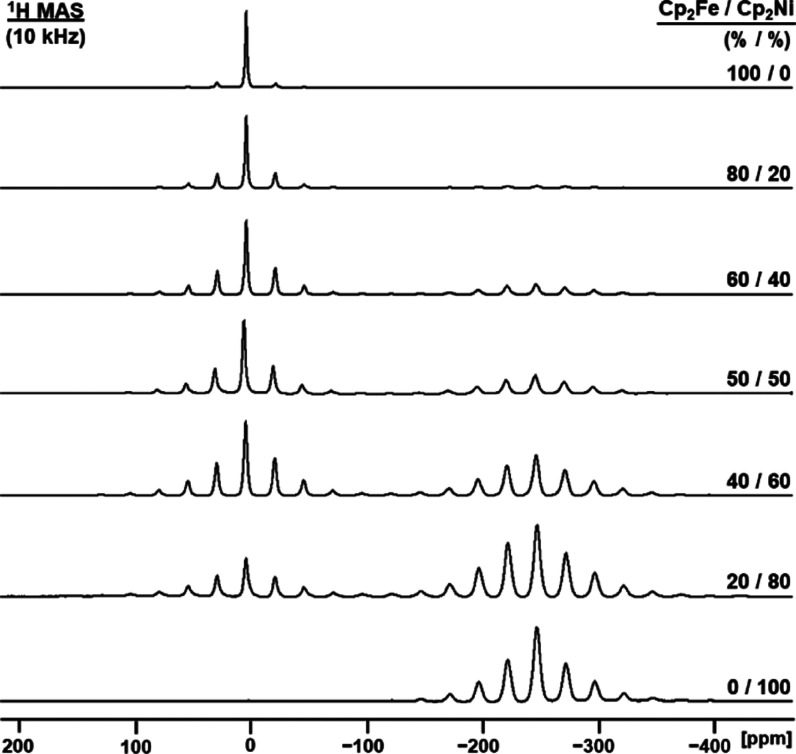
^1^H MAS NMR spectra of solid mixtures of polycrystalline ferrocene (**1**) and nickelocene (**2**) with the indicated molar ratios.

Generally, in the case of a homogeneous 50/50 mixture of **1** and **2** in the solid solution, all molecules of **1** should be affected by the paramagnetism of **2**. For samples with a lower content of **2**, the narrower signal of undisturbed molecules of **1** could, in principle, be visible besides the broad resonance of molecules **1** that are close to **2**. However, the ^1^H MAS spectra of all mixtures of polycrystalline **1** with different amounts of **2** only show one ferrocene and one nickelocene signal (Figure 4). At a 10 kHz rotational speed, the isotropic line of pure polycrystalline ferrocene at about 4 ppm dominates the spectrum (Figure 4, top trace). With increasing nickelocene content, the rotational sidebands of the signal of **1** grow continuously and its overall width increases accordingly. On the other hand, the nickelocene signal at −245 ppm grows in intensity with increasing content of **2** in the sample, but the signal shape practically stays the same.

**Figure 5:**
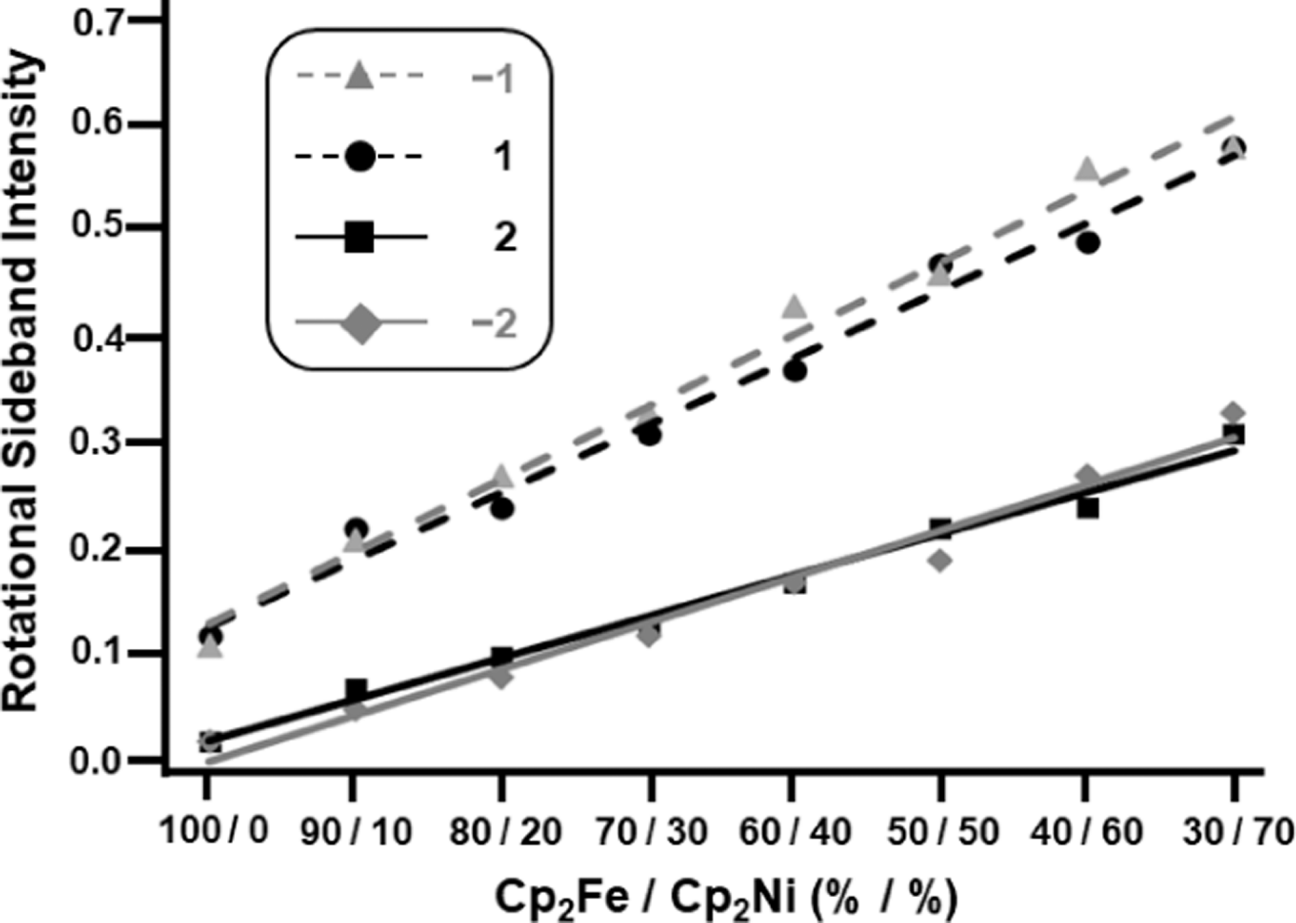
Trendlines produced by applying linear regression to the intensities of first- and second-order rotational sidebands of the ferrocene (**1**) ^1^H MAS signal in solid mixtures with nickelocene (**2**) at the indicated molar ratios. The slopes are 0.0039 and 0.0043 for down- and upfield second-order sidebands and 0.0063 and 0.0068 for the corresponding first-order sidebands.

As mentioned above, unfortunately, the CSA cannot be disentangled from dipolar interactions and BMS effects. Therefore, to describe the change of the ferrocene signal shape with increasing amounts of nickelocene, the intensities of the first- and second-order sidebands have been determined. Table S4 summarizes the data for the signal of ferrocene obtained from samples with the indicated ratios of **1** and **2** in the polycrystalline material. All sideband intensities of **1** increase when more paramagnetic **2** is present. For example, the upfield first-order rotational sideband intensity (*I*_–1_) of the signal of **1** increases from 0.11 to 0.64 (isotropic line set to 1.00) when the nickelocene content increases from 0 to 90% (Table S4). Interestingly, all increases are linear (Figure 5), with the first-order rotational sidebands showing a steeper ascent (slopes of the trendlines 0.0063 and 0.0068) than the second-order sidebands (0.0039 and 0.0043). In practical terms, this shows that the overall signal shape of **1** remains fairly symmetric, independent of the nickelocene content. The sideband intensity data for **2** are reported in Table S5. For the signal of **2** all sideband intensities remain within a comparatively narrow range, for example, going from 0.75 to 0.59 for the first-order upfield sideband (*I*_–1_) when increasing the nickelocene content from 10 to 90%. This shows that the impact of neighboring ferrocene molecules on nickelocene is limited.

Proton NMR signals are usually dominated by dipolar proton–proton interactions in the solid state, and therefore the half-width of the wideline signals of static samples could potentially contribute to the understanding of the broadening of the resonances of **1** in the presence of **2**. Unfortunately, the signal of ferrocene overlaps with the proton background of the probe head. This makes determining the half-width after background subtraction and deconvolution less precise. Nevertheless, proton wideline spectra provide valuable information because they allow for the differentiation of different species with the same chemical shift when narrow signals reside on top of broad resonances. This has been demonstrated for polymers containing water molecules with different mobilities in certain domains.^15,16^ The wideline signals of mixtures of polycrystalline **1** and **2** are displayed in Figure S5, and the values for the half-widths are summarized in Table S6. Although the data scatter somewhat due to the signal widths, as well as background and baseline correction issues, the impact of the paramagnetic **2** on the signal linewidth of **1** is still remarkable. This can be seen when comparing, for example, the signal of pure ferrocene (13 kHz) with that of **1** in a 40/60 mixture of **1**/**2** (27 kHz).

**Figure 6:**
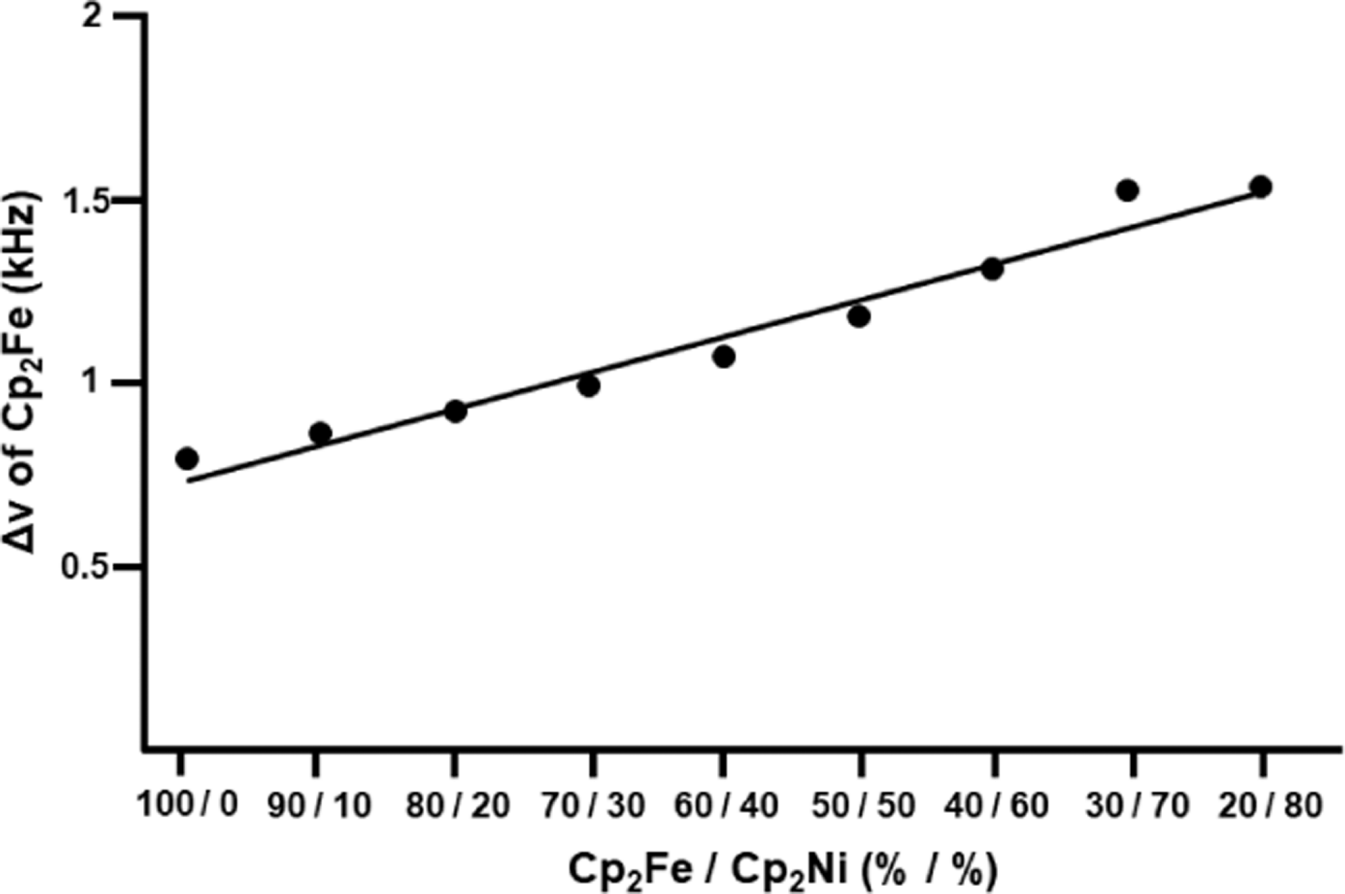
Linear correlation between the residual half-widths of the ferrocene signals in the ^1^H MAS NMR spectra and the nickelocene content in solid mixtures of polycrystalline ferrocene (**1**) and nickelocene (**2**) with the indicated molar ratios.

For samples rotated at high spinning speeds, 10 kHz in this case, the probe head proton background becomes more tractable and, after subtraction, no longer poses a problem for determining the residual linewidths of all signals (Figure 4 and Table 1). The residual linewidth of the ^1^H MAS signal of **1** increases linearly from 0.8 kHz for pure ferrocene to 1.5 kHz for a 20/80 mixture of **1** with **2** (Figure 6). On the other hand, the residual linewidth for the ^1^H MAS resonance of **2** stays practically the same (2.5 kHz) within the margins of error. Overall, the residual ^1^H NMR linewidths are comparatively small, which indicates that the material remains crystalline and does not become amorphous. The crystalline nature of the mixed nickelocene/ferrocene samples is in accordance with the X-ray diffraction result described above which proved that the crystal lattice stays intact and retains the space group *P*2_1_/*n* in a 50/50 mixed crystal.^26^

In principle, it could be considered that the paramagnetic nickelocene changes the overall susceptibility of the sample and in this way broadens the ferrocene signals without necessarily mixing within the crystal lattice on the molecular level. However, placing nickelocene and an equal amount of polycrystalline ferrocene into one rotor and separating both components by a KBr barrier results in signals for **1** and **2** that are identical to their resonances as pure, polycrystalline materials (Figure S6). Therefore, it can be concluded that broadening of the ferrocene signal only takes place when **1** is brought into direct contact with **2** on the molecular level.

Next, we sought to investigate the impact of the paramagnetic nickelocene on the *T*_1_ NMR relaxation time of the signal of diamagnetic **1**. When mixed crystals of **1** and **2**, grown from 50/50 solutions are measured, all ferrocene molecules experience a decrease in their relaxation time. While pure polycrystalline **1** has a *T*_1_ relaxation time of 13.7 s at 10 kHz rotational speed, a 50/50 solid solution of **1** and **2** results in fast relaxation of the ^1^H MAS signal of **1** with a short *T*_1_ time of 4 ms (0.3 ms for **2**). Only one signal of **1** with this fast spin lattice relaxation is found, and this resonance exhibits exponential behavior when subjected to inversion recovery. This means that there are no domains of pure ferrocene left in the crystals and genuine solid solutions with randomly distributed **1** and **2** are generated by crystallizing from solution.

Dissolving ferrocene and nickelocene in a 1:1 ratio and removing the solvent yields polycrystalline material that exhibits the same behavior as the crystals grown from solution. The ferrocene *T*_1_ time has been determined by inversion-recovery experiments (4 ms), as well as the *T*_1_ time of **2** in the material (0.3 ms). Again, only one signal with the same short relaxation time and exponential behavior as the solid solution crystals grown from solutions is found. This means that just removing the solvent from mixtures of **1** and **2** dissolved together leads to solid solutions of the metallocenes with homogeneous distribution of the metal centers. Therefore, the same result is obtained by this fast method as with growing crystals from the dissolved mixtures.

Investigating the materials obtained by dry grinding of components **1** and **2** in different ratios yielded the following results. The *T*_1_ relaxation time of pure polycrystalline **2** is short (0.23 ms). No changes in this relaxation time occur when **2** is diluted by **1** within the error margin. All relaxation data for **1** resulting from the different mixtures obtained by dry grinding are summarized in Table 2. There are two distinct incarnations of ferrocene, as visible in the different inversion-recovery ^1^H NMR spectra (Figure S7) that have been processed using a bi-exponential function (Figure S8). One signal with a long relaxation time in the range of 14–18 s is present. This stems most probably from ferrocene molecules in larger domains which are not impacted by **2**. The other signal of **1**, however, experiences paramagnetic relaxation that is conveyed by dipole–dipole interactions with close-by **2**. Increasing amounts of **2** in the mixed crystals lead to shorter *T*_1_ times for these ferrocene molecules in the vicinity of **1**, changing over one order of magnitude from 3 ms for a 90/10 mixture to 0.2 ms for a 10/90 mixture of **1**/**2** (Table 2).

**Table 2:** ^1^H MAS *T*_1_ Relaxation Times of Ferrocene (**1**) in a Solid Mixture of the Polycrystalline Components with the Indicated Molar Ratios of Ferrocene to Nickelocene (**2**)^a^

ratio **1**/**2** (%)	*T*_1_ of **1** (long) (s)	*T*_1_ of **1** (short) (ms)
100/0	14	
90/10	16	3
80/20	18	2
70/30	17	3
60/40	14	2
50/50	17	2
40/60	19	2
30/70	18	2
20/80	17	0.3
10/90	17	0.2
0/100		

a The spinning speed was 10 kHz, and the individual *T*_1_ values have been obtained using an inversion-recovery technique.

The presence of two distinguishable sorts of ferrocene molecules in ground mixtures can be interpreted by considering the inside of the mixed crystals obtained without grinding (Figure 2). Ferrocene molecules in larger domains of undisturbed sections correspond to those with long *T*_1_ relaxation times. Ferrocene molecules close to the nickelocene pathways would mix on the molecular level and display fast relaxation as a result of their close proximity to the paramagnetic centers. Therefore, it can be concluded that manually grinding the polycrystalline components **1** and **2** (50/50) leads to crystalline material that is not yet a completely homogeneous solid solution, as it was obtained by crystallizing from solvents. However, there is more interaction between ferrocene and nickelocene on the molecular level than in the case of nickelocene migrating into ferrocene single crystals without external manipulation.

So far, all data suggest that whenever nickelocene is present in a solid solution crystal, even in small amounts, the ferrocene molecules in the vicinity of **2** experience the impact of its paramagnetism. To exclude that different, smaller signals may be obscured by the most intensive line, and to avoid a ^1^H background, ^2^H NMR^11,15,16,36,37^ of deuterated ferrocene **1-*****d***_**2**_ ((C_5_H_4_D)_2_Fe) has been employed. An additional bonus is the fact that for paramagnetic metallocenes, the ^2^H NMR signal is much narrower than the ^1^H signal.^24^ The ^2^H MAS spectrum of a 50/50 mixture of polycrystalline **1-*****d***_**2**_ and **2**, prepared by dry grinding of both components, shows that there is limited impact on the Pake pattern of **1-*****d***_**2**_ (Figure S9).^12,14^ Most importantly, no signal of deuterated nickelocene at about −245 ppm is present. Therefore, it can be concluded that **2** migrates as a whole molecule into the crystal lattice of **1**. Any movement of metal atoms through a lattice of static cyclopentadienyl ligands, in other words ligand scrambling, can be excluded. Furthermore, the high mobility observed for deuterated ferrocene molecules adsorbed on silica surfaces and the ensuing collapse of the Pake pattern^12^ can be excluded in the case of the solid solutions of **1** and **2**. The movement of the metallocenes through the crystal lattice appears to be slow and will be quantified in a future project.

## Conclusions

In this study, we could for the first time demonstrate that solid solutions of two metallocenes can be formed by precipitating or crystallizing the components from solutions. The same ratios of ferrocene and nickelocene that had been dissolved were present in the crystals. ^1^H *T*_1_ relaxation time measurements in the solid state proved that for a 50/50 mixture, only one sort of ferrocene molecules is present in the crystal. All ferrocene molecules are in the vicinity of paramagnetic nickelocene because one common short relaxation time is found. Furthermore, it is described for the first time that exposing large ferrocene single crystals to polycrystalline nickelocene leads to the migration of nickelocene into the ferrocene crystal lattice in the absence of a solvent. This observation has been visualized by cutting the single crystals and exposing a marbled pattern of dark green nickelocene within the orange ferrocene crystals. Albeit more slowly, the comparatively volatile nickelocene also migrates into the ferrocene crystals without physical contact through an inert gas atmosphere. The process can be accelerated by bringing the polycrystalline components into contact and grinding them together. Again, no solvent is involved in this process, while the solid solutions are created within minutes. The vicinity of paramagnetic nickelocene to ferrocene on the molecular scale has been successfully probed by solid-state NMR spectroscopy. A comprehensive ^1^H and ^2^H solid-state NMR study of the solid solutions containing different ratios of paramagnetic nickelocene and diamagnetic ferrocene has provided important insights. The signal characteristics and ^1^H *T*_1_ relaxation times of mixed crystals obtained from solutions confirm that ferrocene and nickelocene mix on the molecular level. X-ray diffraction analysis of a mixed 50/50 single crystal of nickelocene and ferrocene proves that the crystal lattice stays intact and retains its space group *P*2_1_/*n*. Residual domains of pure ferrocene in crystals obtained by dry grinding, undisturbed by paramagnetic nickelocene molecules, manifest in the presence of a second, longer *T*_1_ relaxation time. As a first step toward elucidating the migration process, it has been investigated whether the individual metallocenes stay intact or whether the metal centers migrate through a lattice of stationary Cp ligands. For this purpose, deuterated ferrocene has been mixed with nickelocene. ^2^H solid-state NMR did not show a paramagnetic signal for deuterated nickelocene, which would indicate a scrambling of the cyclopentadienyl ligands. Therefore, it can be concluded that the metallocenes migrate within the crystals as whole molecules.

In future projects, the dynamics of the migration of nickelocene through the ferrocene crystal will be studied. Higher temperatures, but staying below the melting points of the components, will be applied during the grinding process. Furthermore, the time dependence of the distribution of nickelocene within the ferrocene crystal lattice will be monitored visually and by *T*_1_ time determinations. Based on quantitative inversion-recovery methods, the ratio of undisturbed ferrocene and ferrocene in the vicinity of nickelocene will be determined. Measurements of temperature-dependent *T*_1ρ_ times of different ratios of ferrocene and nickelocene in mixed crystals will provide insight into domain sizes, as described for polymers.^38^ The effect of a mechanochemical approach, using a ball mill, on the time requirement for obtaining homogeneous solid solutions will be studied. Finally, grinding other paramagnetic and diamagnetic metallocenes together will be probed for the formation of solid solutions.

The fundamental research presented is of interest for solid-state NMR analyses of materials and surfaces in general^39^ and in particular for dynamic nuclear polarization (DNP) methods that involve paramagnetic species.^40^ Furthermore, the solid battery field might profit from the presented investigations of solid-solid mobility. Importantly, the study on organometallic solid solutions has great potential with respect to catalysis and the creation of heterobimetallic nanoparticles with well-defined metal ratios and dual-atom catalysts supported, for example, on silica.

## Supporting Information Available

Additional ^1^H and ^2^H solution and solid-state NMR spectra, half-widths, chemical shifts, *T*_1_ relaxation times, melting point data, and photographs of experimental setups (PDF)
