# Supplementary material for: Creating Solid Solutions of Metallocenes: Migration of Nickelocene into the Ferrocene Crystal Lattice in the Absence of a Solvent
Source: J Phys Chem C Nanomater Interfaces. 2023 Feb 6;127(6):3059–66. doi: 10.1021/acs.jpcc.2c07441 (PMC10848251; doi:10.1021/acs.jpcc.2c07441)
Supplement: Supplementary file 1 — jp2c07441_si_001.pdf [file jp2c07441_si_001.pdf]

Supplementary Information (SI)

Creating Solid Solutions of Metallocenes: Migration of Nickelocene into the Ferrocene Crystal Lattice in the Absence of a Solvent

Gabrielle E. Harmon-Welch, John C. Hoefler, Martha R. Trujillo, Nattamai Bhuvanesh, Vladimir I. Bakhmutov*, Janet Blümel*

Department of Chemistry, Texas A&M University, College Station, TX, 77842-3012, USA.
Email: bluemel@tamu.edu

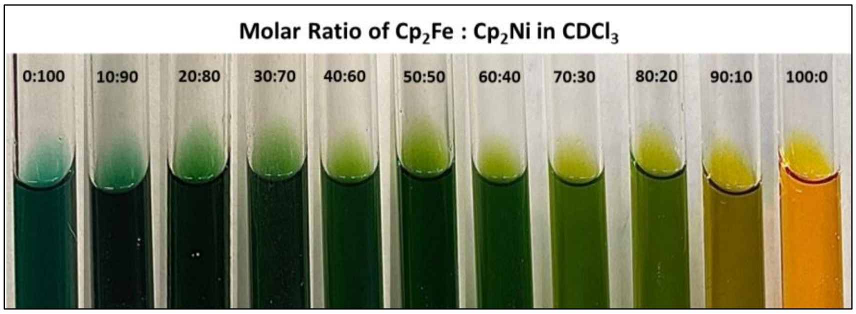

Figure S1. Mixtures of Cp_2Fe (**1**) with Cp_2Ni (**2**) at the indicated ratios, dissolved in $CDCl_3$.

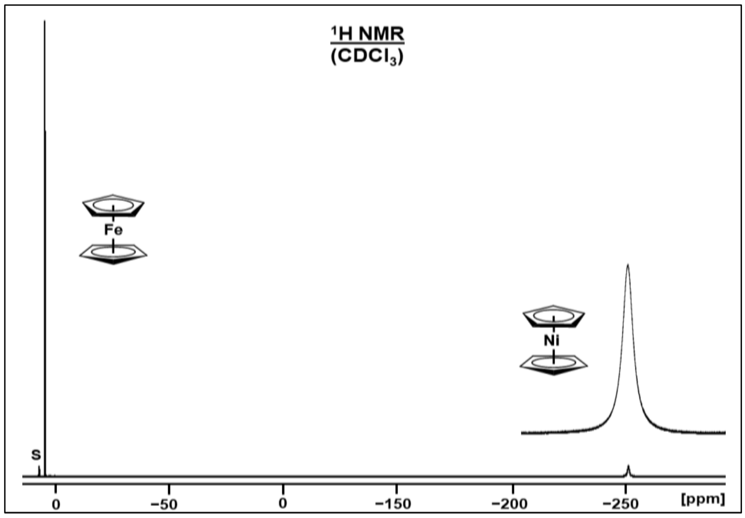

Figure S2. Solution ^1H NMR spectrum of a mixture of 30% Cp_2Fe (**1**) with 70% Cp_2Ni (**2**) dissolved in $CDCl_3$ (signal of residual solvent protons denoted by S).

Table S1. Solution ^{1}H NMR chemical shifts $\delta(^1H)$ and signal halfwidths $\Delta\nu_{1/2}$ of the resonances of ferrocene (**1**) and nickelocene (**2**) dissolved together in $CDCl_3$ at the indicated ratios. All spectra were acquired with 8 scans using a 1 s pulse repetition rate. The chemical shifts were calibrated using the residual proton signal of the solvent (7.24 ppm).

Ratio of 1:2 (mol%)	1		2	
	$\delta(^1H)$ (ppm)	$\Delta\nu_{1/2}$ (Hz)	$\delta(^1H)$ (ppm)	$\Delta\nu_{1/2}$ (Hz)
100 / 0	4.16	2.0	–	–
90 / 10	4.24	2.2	−252.6	543
80 / 20	4.30	2.3	−252.6	523
70 / 30	4.32	2.8	−252.6	557
60 / 40	4.47	3.4	−252.4	535
50 /50	4.54	4.2	−252.3	533
40 / 60	4.59	5.1	−252.3	532
30 / 70	4.69	4.8	−252.2	531
20 / 80	4.80	5.5	−252.2	530
10 / 90	4.90	5.5	−252.0	542
0 / 100	–	–	−252.3	533

Table S2. Solution ^{1}H NMR T_1 relaxation times of the signals of ferrocene (**1**) and nickelocene (**2**) dissolved together in $CDCl_3$ at the indicated ratios. The relaxation times were obtained using an inversion recovery technique.

Ratio 1 / 2 (% / %)	T_1 of 1 (s)	T_1 of 2 (ms)
100 / 0	4.90	-
90 / 10	1.40	0.95
80 / 20	0.84	0.94
70 / 30	0.72	0.95
60 / 40	0.39	0.95
50 / 50	0.32	0.96
40 / 60	0.29	0.96
30 / 70	0.24	0.96
20 / 80	0.19	0.96
10 / 90	0.18	0.96
0 / 100	-	0.96

Table S3. Melting points of polycrystalline ferrocene and nickelocene, as well as the melting range of a 1 : 1 co-crystal of ferrocene and nickelocene. The measurement was performed three times for nickelocene because the value differed from the one provided in the literature. All melting point determinations were carried out using melting point capillaries closed in an inert gas atmosphere.

Ratio of 1:2 (mol%)	Onset pt. (°C)	Single pt. (°C)	Clear pt. (°C)
100 / 0	169.3	172.9	174.0
90 / 10	153.7	161.7	171.5
80 / 20	136.4	144.8	170.4
70 / 30	134.6	142.2	164.9
60 / 40	132.8	143.1	163.6
50 / 50	129.1	140.7	160.8
40 / 60	129.4	142.5	159.1
30 / 70	127.9	134.5	157.8
20 / 80	130.2	139.8	155.2
10 / 90	122.1	129.5	154.0
0 / 100	147.7	151.7	156.3

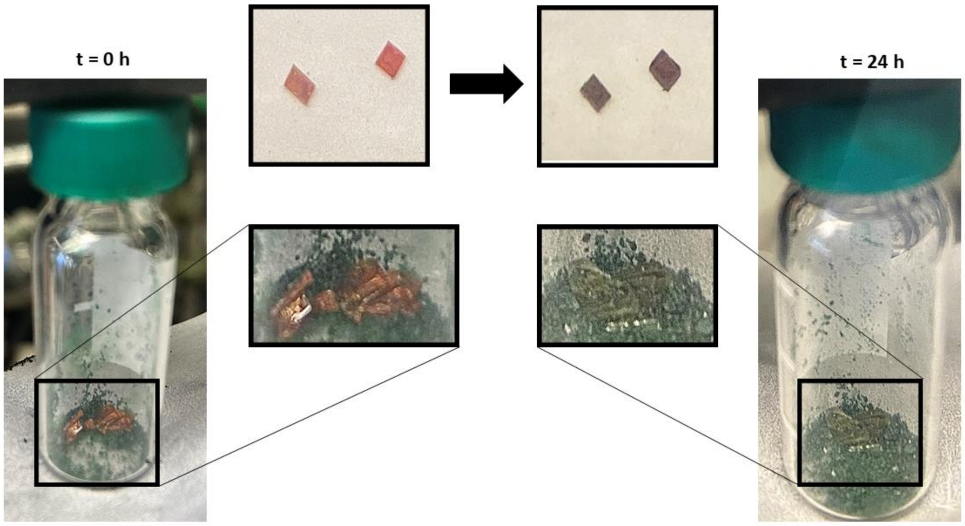

Figure S3. Polycrystalline nickelocene (**2**) migrating into ferrocene (**1**) single crystals. The mixture was allowed to sit in the vial over a time period of 24 h under an inert gas atmosphere. The top photos show the color change that two individual specimens of **1** underwent in the process.

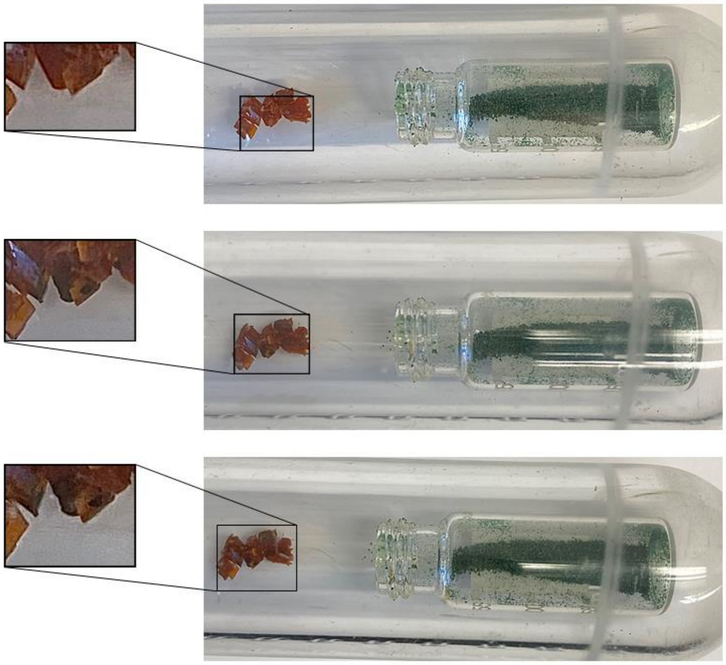

Figure S4. Nickelocene (**2**) migrating into ferrocene (**1**) single crystals through an inert atmosphere in the course of three weeks.

Table S4. Solid-state NMR: Intensities of the first and second order rotational sidebands of the ^{1}H MAS NMR signal of ferrocene (**1**) in the indicated mixtures. The intensities were measured with respect to the isotropic line set to intensity 1.00. The spectra were recorded at 10 kHz rotational frequency. The downfield sidebands of first and second order are labelled I_1 and I_2, the sidebands on the upfield side I_{-1} and I_{-2}. The intensities have been determined after background subtraction.

**Ratio 1 / 2 (% / %)**	**Rotational sideband intensities of Cp_2Fe (1)**			
	**I_2**	**I_1**	**I_{-1}**	**I_{-2}**
100 / 0	0.02	0.12	0.11	0.02
90 / 10	0.07	0.22	0.21	0.05
80 / 20	0.10	0.24	0.27	0.08
70 / 30	0.13	0.31	0.33	0.12
60 / 40	0.17	0.37	0.43	0.17
50 / 50	0.22	0.47	0.46	0.19
40 / 60	0.24	0.49	0.56	0.27
30 / 70	0.31	0.58	0.58	0.33
20 / 80	0.41	0.61	0.51	0.31
10 / 90	0.29	0.54	0.64	0.34
0 / 100	–	–	–	–

Table S5. Solid-state NMR: Intensities of the first and second order rotational sidebands of the ^{1}H MAS NMR signal of nickelocene (**2**) in the indicated mixtures. The intensities were measured with respect to the isotropic line defined as intensity 1.00. The spectra were recorded at 10 kHz rotational frequency. The downfield sidebands of first and second order are labelled I_1 and I_2, the sidebands on the upfield side I_{-1} and I_{-2}. The intensities have been determined after background subtraction.

**Ratio 1 / 2 (% / %)**	**Rotational sideband intensities of Cp_2Ni (2)**			
	**I_2**	**I_1**	**I_{-1}**	**I_{-2}**
100 / 0	–	–	–	–
90 / 10	0.39	0.74	0.75	0.42
80 / 20	0.54	0.88	0.74	0.41
70 / 30	0.49	0.83	0.76	0.41
60 / 40	0.46	0.85	0.71	0.41
50 / 50	0.42	0.77	0.72	0.44
40 / 60	0.41	0.77	0.66	0.36
30 / 70	0.43	0.79	0.64	0.37
20 / 80	0.38	0.76	0.61	0.31
10 / 90	0.34	0.68	0.59	0.31
0 / 100	0.23	0.56	0.51	0.25

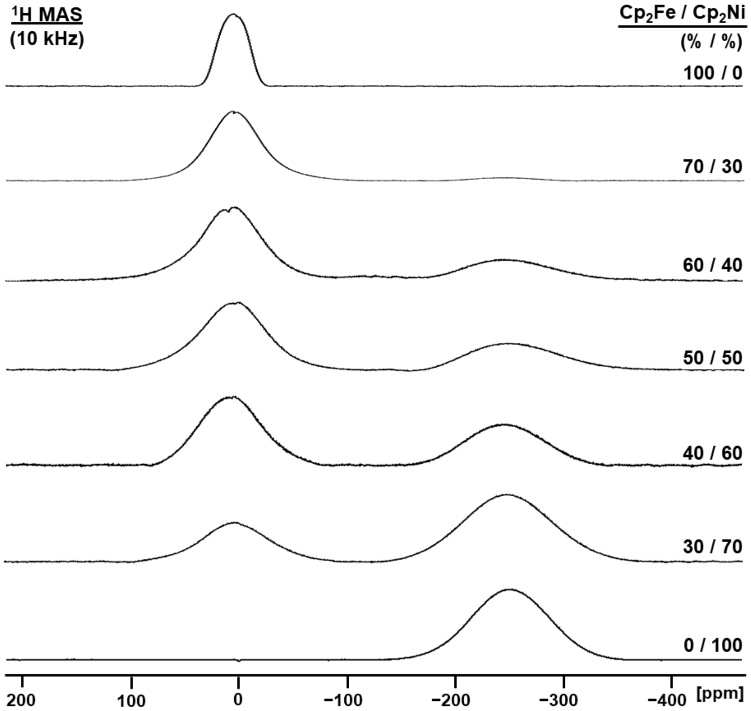

Figure S5. Solid-state NMR: ^{1}H Wideline NMR spectra of polycrystalline **1** and **2** mixed at the indicated molar ratios. The probehead background signal has been subtracted.

Table S6. Solid-state NMR: ^{1}H Wideline NMR signal halfwidths $\Delta\nu_{1/2}$ of ferrocene (**1**) and nickelocene (**2**) in a mixture of the polycrystalline components with the indicated molar ratios. The probehead background signal has been subtracted prior to the linefitting procedure.

Ratio 1 / 2 (% / %)	$\Delta\nu_{1/2}$ of 1 (kHz)	$\Delta\nu_{1/2}$ of 2 (kHz)
100 / 0	13.3	-
90 / 10	15.0	28.8
80 / 20	21.7	30.5
70 / 30	22.0	31.0
60 / 40	24.8	31.1
50 / 50	25.5	31.0
40 / 60	26.9	33.1
30 / 70	26.6	34.4
20 / 80	23.7	34.7
10 / 90	21.6	34.2
0 / 100	-	34.3

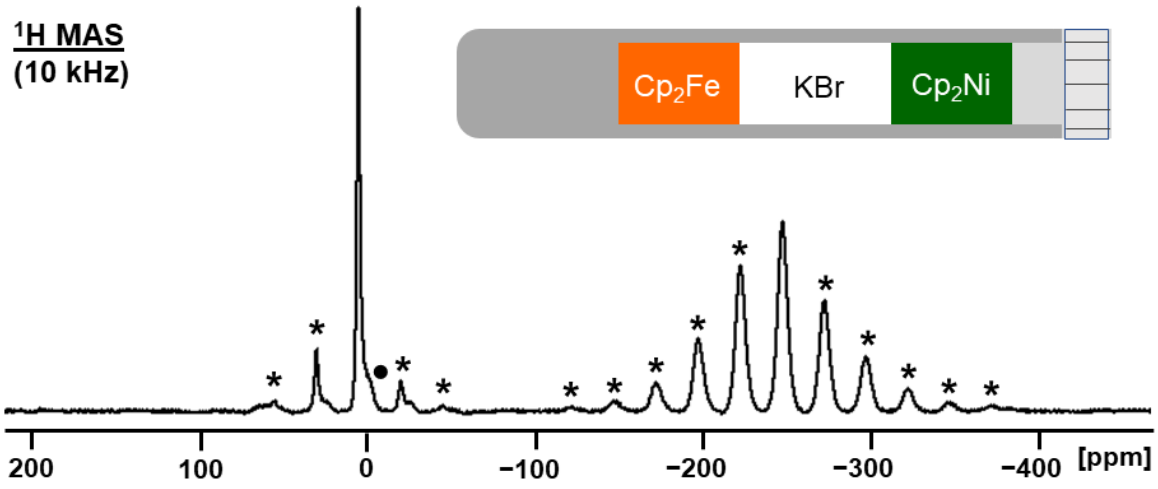

Figure S6. Solid-state NMR: ^{1}H MAS NMR spectrum of polycrystalline **1** and **2**, spatially separated by a layer of KBr within the same HRMAS rotor as depicted schematically. Asterisks denote rotational sidebands, the black dot indicates a trace of water from KBr.

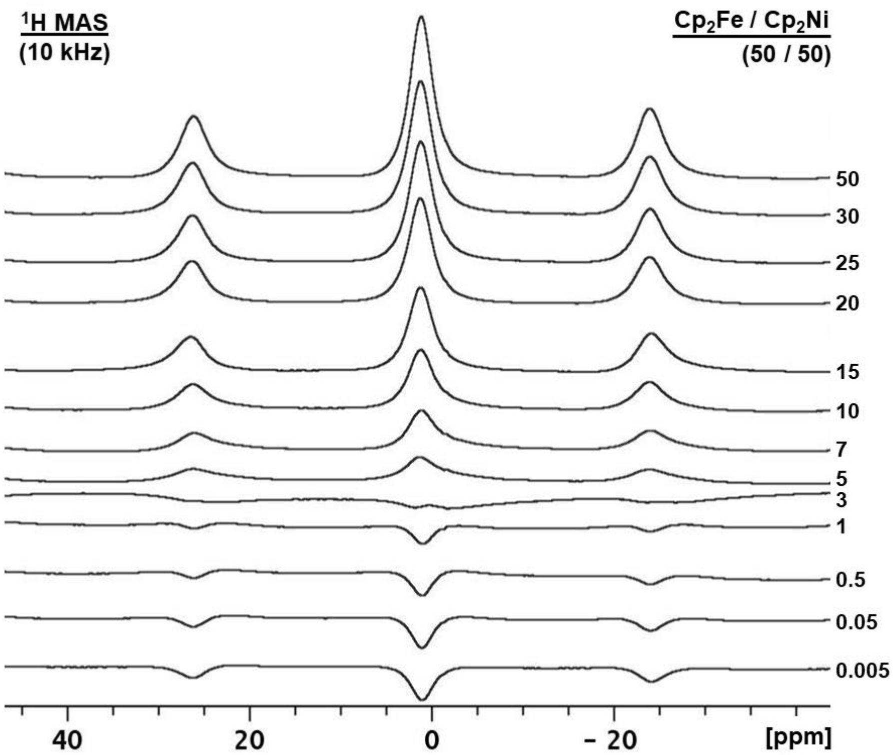

Figure S7. Solid-state NMR: Inversion-recovery ^{1}H MAS NMR spectra obtained at a spinning rate of 10 kHz for the dry ground mixture ferrocene/nickelocene 50/50 with the indicated τ delays [s].

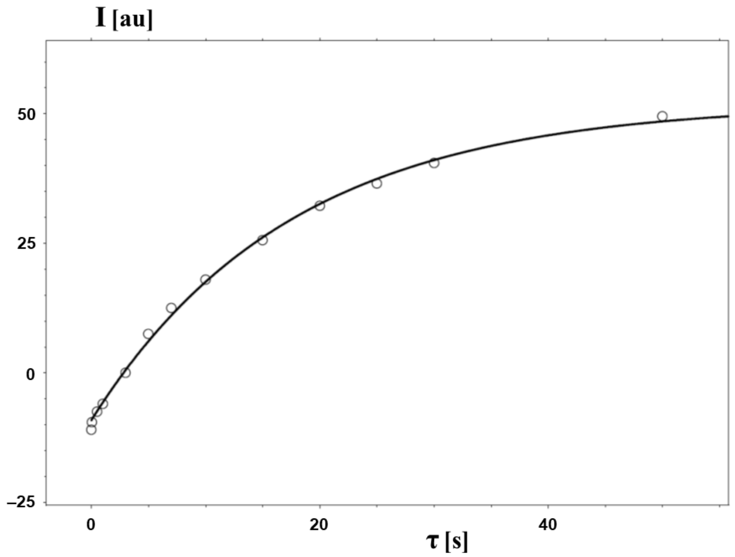

Figure S8. Solid-state NMR: Experimental inversion−recovery curve obtained for the dry-ground mixture ferrocene/nickelocene 50/50 after fitting to a bi-exponential function.

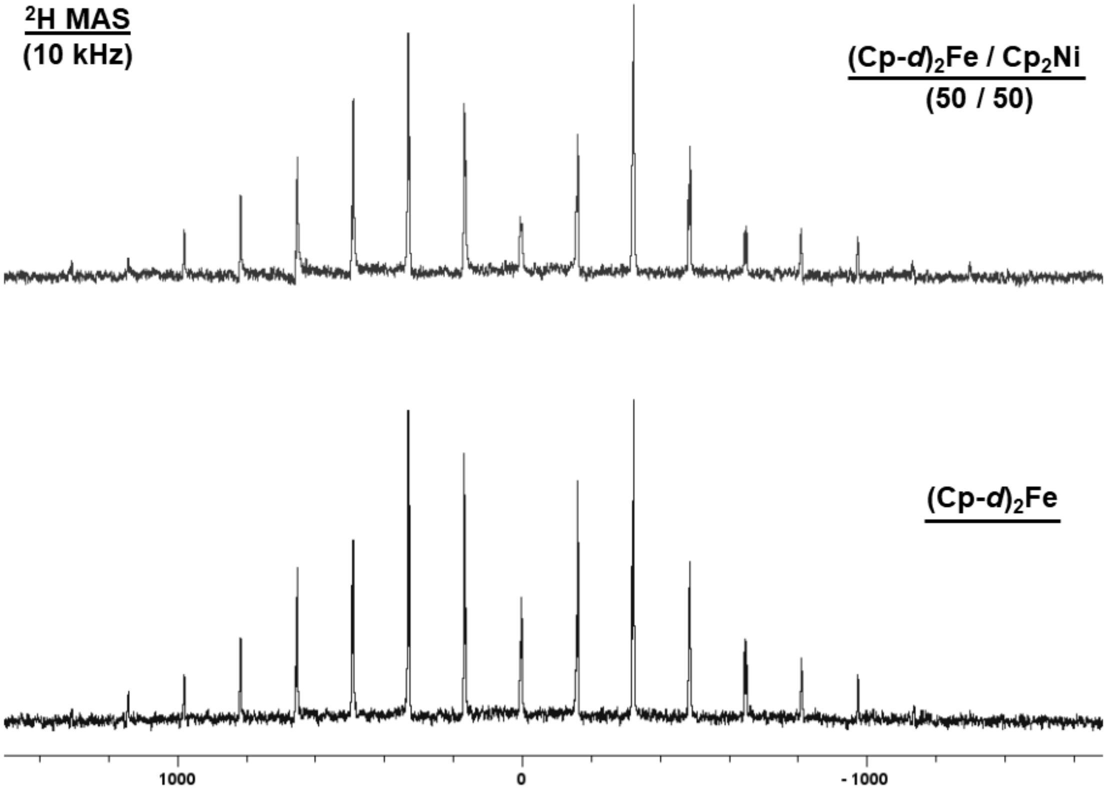

Figure S9. Solid-state NMR: ^{2}H MAS NMR spectra of polycrystalline pure deuterated ferrocene (bottom) and a sample of deuterated ferrocene dry-ground with nickelocene (50/50).
